# Supplementary material for: Constraint of Base Pairing on HDV Genome Evolution
Source: Viruses. 2021 Nov 23;13(12):2350. doi: 10.3390/v13122350 (PMC8708965; doi:10.3390/v13122350)
Supplement: Supplementary file 1 [file viruses-13-02350-s001.zip › SupplementaryData4.pdf]

Supplementary Data S4

Constraint of Base-Pairing on HDV Genome Evolution

by Nagata, S., Kiyohara, R., and Toh, H.

Summary of Evolutionary Distances

The minimum, median, mean and maximum of the five evolutionary distances are shown for each comparison.

within Genotype I

	min	median	mean	max
$ed_{\mathrm{bp}}(x, y)$	0.0	3.7×10^{-2}	3.8×10^{-2}	1.4×10^{-1}
$ed_{\mathrm{nbp}}(x, y)$	9.8×10^{-3}	7.8×10^{-2}	8.2×10^{-2}	2.6×10^{-1}
$ed(x, y)$	5.0×10^{-3}	6.3×10^{-2}	6.5×10^{-2}	1.5×10^{-1}
$K_s^c(x, y)$	1.0×10^{-2}	2.3×10^{-1}	2.4×10^{-1}	6.5×10^{-1}
$K_a^c(x, y)$	0.0	7.0×10^{-2}	7.3×10^{-2}	1.9×10^{-1}

within Genotype II

	min	median	mean	max
$ed_{\mathrm{bp}}(x, y)$	0.0	9.4×10^{-2}	9.0×10^{-2}	2.1×10^{-1}
$ed_{\mathrm{nbp}}(x, y)$	9.5×10^{-3}	1.2×10^{-1}	1.2×10^{-1}	3.2×10^{-1}
$ed(x, y)$	2.5×10^{-3}	1.3×10^{-1}	1.2×10^{-1}	2.8×10^{-1}
$K_s^c(x, y)$	1.0×10^{-2}	2.5×10^{-1}	3.0×10^{-1}	7.2×10^{-1}
$K_a^c(x, y)$	0.0	8.0×10^{-2}	1.1×10^{-1}	2.4×10^{-1}

within Genotype III

	min	median	mean	max
$ed_{\mathrm{bp}}(x, y)$	7.5×10^{-3}	3.2×10^{-2}	3.0×10^{-2}	4.8×10^{-2}
$ed_{\mathrm{nbp}}(x, y)$	3.4×10^{-2}	8.7×10^{-2}	8.1×10^{-2}	1.2×10^{-1}
$ed(x, y)$	3.1×10^{-2}	6.4×10^{-2}	5.8×10^{-2}	7.6×10^{-2}
$K_s^c(x, y)$	3.0×10^{-2}	5.0×10^{-2}	7.4×10^{-2}	1.3×10^{-1}
$K_a^c(x, y)$	3.0×10^{-2}	5.0×10^{-2}	5.2×10^{-2}	9.0×10^{-2}

between Genotypes I and II

	min	median	mean	max
$ed_{\mathrm{bp}}(x, y)$	1.3×10^{-2}	1.3×10^{-1}	1.2×10^{-1}	2.3×10^{-1}
$ed_{\mathrm{nbp}}(x, y)$	4.3×10^{-2}	1.7×10^{-1}	1.7×10^{-1}	3.5×10^{-1}
$ed(x, y)$	7.8×10^{-2}	1.7×10^{-1}	1.7×10^{-1}	2.9×10^{-1}
$K_s^c(x, y)$	3.9×10^{-1}	6.2×10^{-1}	6.2×10^{-1}	1.0
$K_a^c(x, y)$	1.4×10^{-1}	1.9×10^{-1}	1.9×10^{-1}	3.6×10^{-1}

between Genotypes I and III

	min	median	mean	max
$ed_{\mathrm{bp}}(x, y)$	2.0×10^{-1}	2.5×10^{-1}	2.5×10^{-1}	3.1×10^{-1}
$ed_{\mathrm{nbp}}(x, y)$	2.5×10^{-1}	4.2×10^{-1}	4.2×10^{-1}	6.6×10^{-1}
$ed(x, y)$	2.9×10^{-1}	3.5×10^{-1}	3.5×10^{-1}	4.2×10^{-1}
$K_s^c(x, y)$	7.3×10^{-1}	9.8×10^{-1}	9.8×10^{-1}	1.3
$K_a^c(x, y)$	2.5×10^{-1}	2.8×10^{-1}	2.9×10^{-1}	3.5×10^{-1}

between Genotypes II and III

	min	median	mean	max
$ed_{\mathrm{bp}}(x, y)$	1.7×10^{-1}	2.3×10^{-1}	2.3×10^{-1}	3.1×10^{-1}
$ed_{\mathrm{nbp}}(x, y)$	2.3×10^{-1}	3.6×10^{-1}	3.7×10^{-1}	5.6×10^{-1}
$ed(x, y)$	2.7×10^{-1}	3.2×10^{-1}	3.2×10^{-1}	3.9×10^{-1}
$K_s^c(x, y)$	6.1×10^{-1}	7.3×10^{-1}	7.3×10^{-1}	9.0×10^{-1}
$K_a^c(x, y)$	2.3×10^{-1}	2.8×10^{-1}	2.8×10^{-1}	3.4×10^{-1}
